# Programming Cell‐Derived Vesicles with Enhanced Immunomodulatory Properties

**DOI:** 10.1002/adhm.202301163

**Published:** 2023-07-09

**Authors:** Khaga R. Neupane, Geraldine S. Ramon, Brock Harvey, Byeong Chun, Surya P. Aryal, Abdullah A. Masud, J. Robert McCorkle, Jill M. Kolesar, Peter M. Kekenes‐Huskey, Christopher I. Richards

**Affiliations:** ^1^ Department of Chemistry University of Kentucky 506 Library Drive, 125 Chemistry‐Physics Building Lexington KY 40506 USA; ^2^ Department of Cell and Molecular Physiology Loyola University Chicago Chicago IL USA; ^3^ Department of Pharmacy Practice and Science College of Pharmacy University of Kentucky Lexington KY 40508 USA

**Keywords:** cancer immunotherapy, macrophages, polarization, signaling, vesicles

## Abstract

Tumor‐associated macrophages are the predominant immune cells present in the tumor microenvironment and mostly exhibit a pro‐tumoral M2‐like phenotype. However, macrophage biology is reversible allowing them to acquire an anti‐tumoral M1‐like phenotype in response to external stimuli. A potential therapeutic strategy for treating cancer may be achieved by modulating macrophages from an M2 to an M1‐like phenotype with the tumor microenvironment. Here, programmed nanovesicles are generated as an immunomodulatory therapeutic platform with the capability to re‐polarize M2 macrophages toward a proinflammatory phenotype. Programmed nanovesicles are engineered from cellular membranes to have specific immunomodulatory properties including the capability to bidirectionally modulate immune cell polarization. These programmed nanovesicles decorated with specific membrane‐bound ligands can be targeted toward specific cell types including immune cells. Macrophage‐derived vesicles are engineered to enhance immune cell reprogramming toward a proinflammatory phenotype.

## Graphical abstract

Cell‐derived vesicles engineered from macrophages are programmed with surface modifications to enhance their immunomodulatory activity. These vesicles demonstrate an increased capability to reprogram targeted macrophages toward a proinflammatory phenotype.

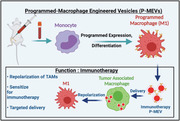

## 1 Introduction

Vesicle‐based nanoparticles including exosomes,^[^ ^1^ ^]^ microvesicles,^[^ ^2^ ^]^ and liposomes^[^ ^3^ ^]^ have been leveraged as potential therapeutic tools for cancer treatment due to their ability to specifically target the tumor environment and their ability to elicit a tumor‐specific immune response. Exosomes are nano‐sized (diameter 40–150 nm) extracellular vesicles (EVs) released by the cells through normal physiological processes^[^ ^4^ ^]^ and contain a wide range of biological cargo including proteins and RNA which can be used to communicate information to target cells.^[^ ^5^ ^]^ Exosome targeting specificity can be harnessed to controllably deliver therapeutics both in cell culture and in vivo. In addition, exosomes released by antigen‐presenting cells (APCs) including dendritic cells and macrophages can also activate the immune system.^[^ ^1^, ^2^ ^]^ Despite these promising characteristics, low production yields and difficulties in separating exosomes from biological solutions still pose barriers to their use in clinical applications.^[^ ^6^ ^]^ Liposomes, synthetically generated lipid bilayer vesicles, have been used as an alternative to exosomes in therapeutic delivery.^[^ ^7^ ^]^ While liposomes can be produced in large quantities, they lack the inherent biocompatibility seen with endogenous exosomes and are prone to immune clearance when delivered in vivo.^[^ ^8^ ^]^ Recently, cell‐derived vesicles (CDVs) obtained by fragmenting cellular membranes have been found to mimic many of the positive attributes of exosomes and have shown promise as therapeutic delivery platforms because they can be produced in high yield, exhibit targeting specificity and have low immunogenicity when delivered in vivo.^[^ ^6^, ^9^ ^]^

Vesicles derived from antigen‐presenting cells offer additional potential avenues for therapeutics because of their ability to serve as immunomodulatory platforms.^[^ ^1^, ^10^ ^]^ Macrophages are the most abundant immune effector cells present in the tumor microenvironment and exhibit a continuum of functional states between pro‐inflammatory (M1) and anti‐inflammatory (M2) polarization.^[^ ^11^ ^]^ M1 macrophages are known to have anti‐tumoral properties including engulfing and destroying phagocytosed tumor cells and activating different components of the immune system. However, M2 macrophages stimulate tumor angiogenesis and inhibit the anti‐tumor immune response mediated by T‐cells.^[^ ^12^ ^]^ Along with small molecule immunomodulators^[^ ^13^ ^]^ and extracellular vesicles,^[^ ^1^ ^]^ cell‐derived vesicles^[^ ^14^ ^]^ from M1 macrophages have been shown to alter the polarization of tumor‐associated macrophages which play a role in chemotherapy resistance and promote metastasis. While these therapeutic approaches show promise, they suffer from unique challenges. The efficacy of small molecule‐based therapeutics is limited by their rapid degradation and inability to preferentially target tumor‐associated macrophages (TAMs) in vivo. While EVs are biostable, exhibit targeting specificity, and can modulate macrophage phenotype in the tumor microenvironment, EV‐based therapies are challenged by their low production yield. Cell‐derived vesicle‐based therapies overcome several challenges that limit other nanoscale therapeutics, but CDVs would be more effective with more specific targeting and higher efficacy in repolarizing anti‐inflammatory macrophages to a proinflammatory phenotype.

**Scheme 1:**
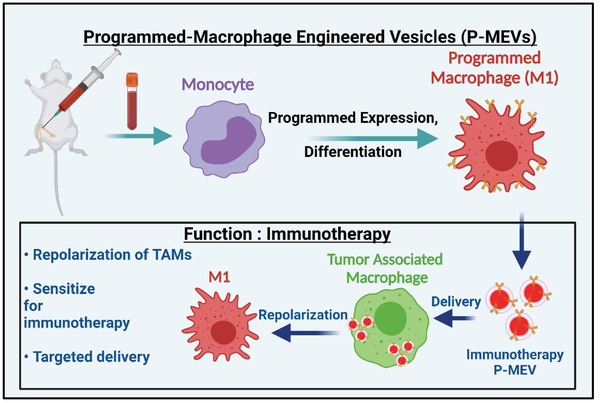
Schematic diagram illustrating the approach of generating programmed MEVs from programmed bone marrow‐derived macrophages. Macrophages (M0) are first programmed to overexpress desired ligands on their surface and then polarized into either pro‐inflammatory macrophages (M1). Programmed MEVs were generated using nitrogen cavitation and MEVs were purified from cellular fragments by serial centrifugation. M2 macrophages were then treated with programmed MEVs to shift their polarization toward the pro‐inflammatory phenotype.

One approach for increasing macrophage targeting and the functionality of immunomodulatory capability of therapeutic delivery systems, such as cell‐derived vesicles, is to functionalize their surface with specific moieties that would endow them with enhanced immunomodulatory and macrophage targeting capabilities. A similar approach has been utilized for synthetic therapeutic‐loaded lipid‐polymer‐based nanoparticles which were engineered with 1,2‐distearoyl‐sn‐glycero‐3‐phosphoethanolamine‐poly(ethylene glycol) (DSPE‐PEG)‐mannose and monophosphoryl lipid A (MPLA) to simultaneously improve their dendritic cell targeting and ability to execute enhanced immune responses.^[^ ^15^ ^]^ Similarly, toll‐like receptor 7 and 8 (TLR7/8) agonists presenting nanoparticles have been generated using poly(ethylene glycol)‐poly(lactic acid) (PEG‐PLA) to enhance the immunomodulatory properties of nanoparticles.^[^ ^16^ ^]^ Here, we developed programmed cell‐derived nanovesicles generated from multiple cell types including macrophages through the targeted over‐expression of specific ligands to achieve greater efficacy in reprogramming anti‐inflammatory macrophages toward a proinflammatory phenotype. Overall, programmed nanovesicles based therapeutics show promise for enhanced ability to modulate immune cell inflammatory phenotypes (**Scheme** **1**).

## 2 Results and Discussion

### 2.1 MEV Characterization

**Figure 1:**
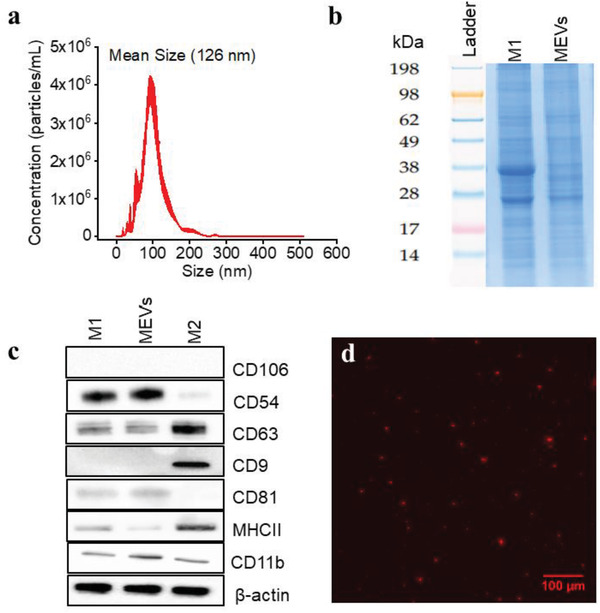
Macrophage Engineered Vesicles (MEVs) Characterization a) Size distribution of MEVs obtained from Nanoparticle Tracking Analysis (NTA). Nitrogen cavitation of the macrophages results in the generation of MEVs of effective diameter between 50–200 nm. b) Sodium dodecyl sulfate‐polyacrylamide gel electrophoresis (SDS‐PAGE) analysis of the protein content of MEVs compared to M1 macrophages. c) Validation of exosome marker proteins in MEVs. Equal amounts of total proteins extracted from MEVs, M1 macrophages, and M2 macrophages were immunoblotted for CD106, CD54, CD63, CD9, CD81, MHCII, and CD11b. d) Wide‐field fluorescence image of MEVs labeled with a lipophilic dye DiI. Scale bar = 100 µm.

We utilized pro‐inflammatory (M1) bone marrow‐derived macrophages (BMDMs) to generate macrophage‐engineered vesicles (MEVs) through the disruption of the cell membrane with nitrogen cavitation. This leads to the formation of nano‐sized membrane fragments that rearrange to form vesicles.^[^ ^17^ ^]^ To characterize vesicle concentration and size distribution, we used nanoparticle tracking analysis (NTA) which allows us to measure both the size distribution and the concentration of the vesicles in solution. We found that 100 million M1 BMDMs generated ≈2 × 10^12^ MEVs. The size distribution of the MEVs obtained from nanoparticle tracking analysis is primarily between 50–200 nm (**Figure** **1**a), which is similar to the range reported for exosomes.^[^ ^10^ ^]^ The mean diameter of MEVs obtained from NTA was 127 nm. We also performed transmission electron microscopy (TEM) which gave similar results in terms of vesicle diameter and size distribution (Figure S1, Supporting Information). To determine the stability of MEVs, we next measured the surface charge of the MEVs using a Malvern Zetasizer. The zeta potential of MEVs was −5.2 mV. This is similar to values seen for endogenously released vesicles such as exosomes which have been reported to have a zeta potential between −5 to −20 mV.^[^ ^18^ ^]^ We next utilized sodium dodecyl sulfate‐polyacrylamide gel electrophoresis (SDS‐PAGE) to determine the proteins present on the surface of MEVs in comparison to the parent M1 macrophage. We observed similar protein bands from MEVs and M1 macrophages indicating that vesicle formation retains most of the same proteins present on the cell surface (Figure 1b). Exosomal marker proteins such as tetraspanins (CD9 and CD63), integrins (CD81, CD82), chaperones heat shock protoeins 60 and 70 (HSP60, HSP70), immunoglobulins (intracellular adhesion molecule 1 (ICAM‐1) and vascular adhesion protein 1 (VCAM1)), and major histocompatibility complex class I (MHC‐I) and II (MHC‐II) have been shown to be present on M1 exosomes and have been implicated in adhesion, signaling, and activation when exosomes are delivered to target macrophages.^[^ ^19^ ^]^ We performed western blotting for several of these exosomal marker proteins including CD9, CD54, CD63, CD81, and CD106 and found that the majority of these proteins including CD54 (ICAM‐1), CD63, MHCII, CD11b, CD81 are present in MEVs (Figure 1c). However, we found that other exosomal marker proteins including CD9, and CD106 were absent in MEVs. Western blotting analysis demonstrated that CD9 and CD106 were also absent from parent M1 BMDMs. Tetraspanins including CD9, and CD63 are integral membrane proteins, embedded within the cellular membranes and have been shown to play a vital role in the fusion of exosomes with target cells.^[^ ^20^ ^]^ Similarly, ICAM‐1 is a transmembrane glycoprotein that has been shown to mediate cell–cell interaction and outside‐in cell signaling during an immune response.^[^ ^21^ ^]^ The presence of a majority of the same surface markers in MEVs indicates that vesicles engineered through nitrogen cavitation likely have similar properties to exosomes.

**Figure 2:**
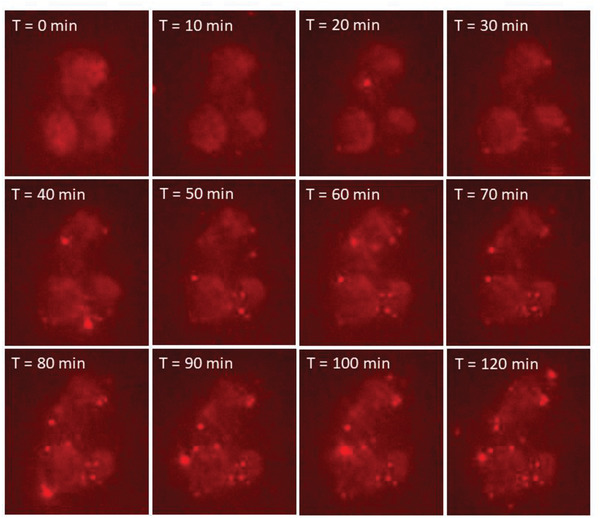
Series of images showing DiI‐labeled MEVs uptake by M2 BMDMs. Vesicles were added after the first time point in real time and images were taken for 2 h every 10 min. A 40× air objective was used with 561 nm excitation. Vesicles were labeled using the lipophilic dye, DiI.

We next performed a set of experiments to see if MEVs could successfully be delivered to M2 BMDMs similar to what has been shown for exosomes.^[^ ^1^, ^10^ ^]^ We first labeled MEVs with 1,1'‐dioctadecyl‐3,3,3',3'‐tetramethylindocarbocyanine perchlorate (DiI), a lipophilic, non‐toxic, fluorescent label that embeds into the membrane of the vesicles. We confirmed the labeling of these vesicles using fluorescence microscopy. The red punctate regions seen in Figure 1d indicate the successful labeling of vesicles with DiI. We used these labeled vesicles in time‐lapse imaging experiments to record vesicle delivery at multiple time points over the course of 2 h by incubating M2 BMDMs with 1 × 10^9^ labeled vesicles. MEV uptake was tracked using the fluorescence intensity of the labeled vesicles in the otherwise unlabeled cells. Images were taken at 10‐minute time intervals for 2 h. We observed a gradual uptake of MEVs by M2 BMDMs (**Figure** **2**) over time as indicated by the increase in fluorescence intensity. These preliminary characterization results suggest that nitrogen cavitation‐generated MEVs have similar size, zeta potential, and surface markers as exosomes and these vesicles are efficiently taken up by M2 BMDMs.

### 2.2 MEV‐Mediated Phenotypic Reprogramming of M2‐BMDMs

**Figure 3:**
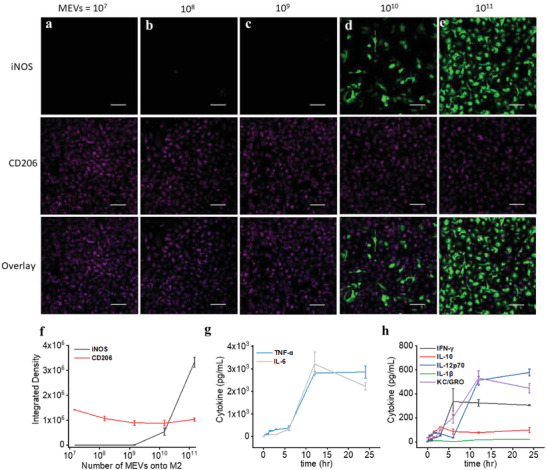
Reprogramming macrophage polarization by MEVs. a–f) Immunostaining of iNOS (M1 macrophage marker) and CD206 (M2 macrophage marker) in M2 macrophages after incubation with 10^8^, 10^9^, 10^10^ and 10^11^ MEVs for 24 h. Each data point is the average of at least 5 experiments (*n* = 5). The data are presented as the mean ± SEM. g,h) Measurement of the proinflammatory cytokines including IFN‐*γ*, IL‐10, IL‐12p60, IL‐1*β*, IL‐6, KC/GRO, and TNF‐*α* released by M2 macrophages after incubating them with 1 × 10^11^ MEVs in a time‐dependent manner. Each data point is the average of at least 3 experiments (*n* = 3). The data are presented as the mean ± SEM.

We next confirmed the ability of pro‐inflammatory (M1) MEVs to reprogram anti‐inflammatory (M2) BMDMs toward a proinflammatory phenotype using a series of cell membrane modifications. We first validated a shift in macrophage phenotype using immunocytochemistry analysis to measure the expression of inducible nitric oxide synthase (iNOS), a pro‐inflammatory macrophage marker, in M2 BMDMs after they were incubated with different concentrations of MEVs derived from M1 cells. We incubated 50 000 M2 BMDMs with an increasing concentration of MEVs ranging from 10^7^ to 10^11^ for 12 h at 37 °C. We observed a clear increase in iNOS for M2 BMDMs upon incubation with increasing concentrations of MEVs (**Figure** **3**a–e). At a concentration of 10^11^ vesicles, we observed a robust expression of iNOS (Figure 3e). These results demonstrate that M2 BMDMs can be reprogrammed through MEV exposure shifting polarization toward a pro‐inflammatory phenotype. We performed additional immunocytochemistry analysis to understand the effect of MEV‐driven M2 to M1‐like polarization on the expression of CD206 an M2 macrophage marker. We observed a gradual decrease in CD206 expression (Figure 3a–f). Overall, these results indicate that at a suitable concentration, MEVs can reprogram M2 BMDMs toward an M1‐like phenotype.

We also performed a time‐dependent repolarization assay for M2 macrophages incubated with MEVs and simultaneously assessed seven different pro‐inflammatory cytokines (IFN‐*γ*, IL‐10, IL‐12p70, IL‐1*β*, IL‐6, KC/GRO, and TNF‐*α*) released by MEV‐treated M2 BMDMs. We added 1 × 10^11^ vesicles to 50 000 macrophages in culture and analyzed cytokine release by MEV‐treated M2 macrophages after 0.5, 1, 1.5, 2, 3, 6, 12, and 24 h of incubation. Compared to the low level of pro‐inflammatory cytokines released by unmodified M2 BMDMs, we observed an increase in cytokine levels as early as 2 h of incubation. As the incubation time progresses, we observe an increase in cytokine release by MEV‐treated M2 BMDMs. Pro‐inflammatory cytokine releases into the supernatant plateaued after 12 h of incubation of M2 BMDMs with MEVs (Figure 3g,h). We did not see differences in the cytokine release by MEV‐treated M2 BMDMs between 12 and 24 h of incubation time. These results indicate that MEV‐mediated M2 to M1 repolarization depends both on the concentration of MEVs and the incubation period.

### 2.3 Role of MEV‐Anchored Endogenous Ligands on M2 to M1 Macrophage Modulation

We hypothesized that proteins anchored within the membrane of MEVs control targeting, cellular uptake, and drive changes in macrophage phenotype. Because MEVs are generated from parent pro‐inflammatory macrophages and maintain proteins resident on the surface of M1 macrophages (Figure 1b), MEVs may carry a wide range of membrane‐bound cytokines, chemokines, transmembrane proteins, and other cell signaling endogenous ligands that belong to the parent macrophage. These proteins likely interact with the surface proteins on the recipient anti‐inflammatory (M2) macrophages initiating signaling cascades that lead to their repolarization toward an M1 phenotype.

We first performed a set of studies to validate that membrane proteins played a role in MEV‐induced macrophage repolarization. It is possible that cytokines, cell‐associated signaling proteins, anchored in the plasma membrane of the cell could be responsible for macrophage reprogramming. For example, pro‐inflammatory cytokines including TNF‐*α*, IFN‐*γ*, and IL‐12 produced by classically activated macrophages can stimulate macrophage polarization toward an M1 phenotype.^[^ ^22^ ^]^ We examined vesicle solutions for the presence of seven pro‐inflammatory cytokines including IFN‐*γ*, IL‐10, IL‐12p70, IL‐1*β*, IL‐6, KC/GRO, and TNF‐*α* to determine if residual cytokines contributed to MEV‐mediated M2 to M1 repolarization. To analyze the cytokines entrapped in the interior of MEVs, we freeze‐fractured MEVs to rupture them and released the entrapped cargo from inside of the vesicles. Cytokine assays showed that MEVs contain low levels of pro‐inflammatory cytokines in the interior of the vesicles and virtually no cytokines in the vesicle solution (Figure S2a, Supporting Information). We further investigated if the concentration of cytokines present in the vesicle suspension was sufficient to reprogram M2 macrophages toward an M1 phenotype. We performed an IFN‐*γ* dose response with M2 macrophages and assessed tumor necrosis factor alpha (TNF‐*α)* released by M2 macrophages into the supernatant after treatment (Figure S2c, Supporting Information). Results showed that the small amount of cytokines present in MEVs (≈40 pg mL^−1^) was not capable of altering the M2 macrophage phenotype and had a negligible contribution to mediating M2 to M1 repolarization (Figure S2b, Supporting Information).

**Figure 4:**
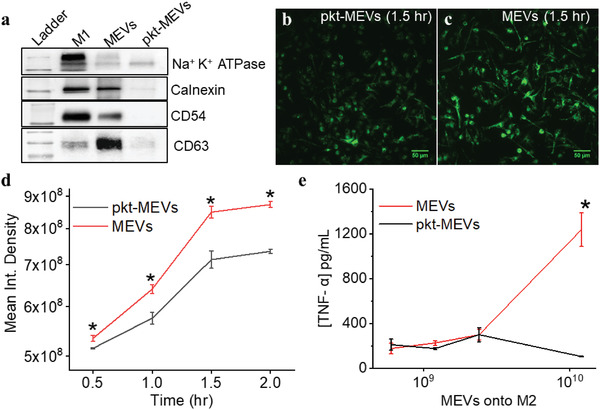
Proteolytic digestion of membrane proteins present on MEVs eliminates the reprogramming capability of M2 macrophages toward an M1 phenotype. a) Western blotting to compare the presence of membrane‐anchored proteins in MEVs treated with and without proteinase‐K. b) Widefield fluorescence images of DiI labeled proteinase‐K treated MEVs (pkt‐MEVs) and untreated MEVs delivered to M2 macrophages after 1.5 h show the reduction in uptake of proteinase‐K treated MEVs. c,d) Comparison of delivery of fluorescent labeled MEVs (red) and fluorescent labeled pkt‐MEVs (black) to M2 macrophages. e) TNF‐*α* is released by M2 macrophages in a dose‐dependent manner after 24 h of interaction with increasing concentrations of MEV or pkt‐MEVs. Each data point is the average of at least 3 experiments (*n* = 3). The data is presented as the mean ± SEM. A Two‐Sample *t*‐Test was used to determine the statistical significance between the endpoints. *p < 0.01 indicates that the results are statistically significant.

We next investigated the importance of ligand‐receptor interactions on the uptake of MEVs by M2 macrophages and MEV‐mediated M2 to M1 macrophage repolarization. For this, we carried out proteolytic digestion of the membrane proteins embedded in the membrane of MEVs using proteinase‐K (0.5 mg mL^−1^). Proteinase‐K is a broad‐spectrum proteolytic enzyme that is commonly used to digest proteins.^[^ ^23^ ^]^ We performed a western blotting analysis to confirm the elimination of membrane‐anchored proteins present on the surface of MEVs after proteinase‐k digestion. We compared the expression of Na^+^K^+^ ATPase (a plasma membrane marker), calnexin (an endoplasmic reticulum marker), CD54 (a transmembrane glycoprotein), and CD63 (a transmembrane protein) in M1 macrophages (control), MEVs, and proteinase‐K‐treated MEVs. We found that proteinase‐K treatment of MEVs resulted in nearly the complete digestion of the membrane proteins that we analyzed (**Figure** **4**a). We then performed a set of experiments to compare the delivery of purified proteinase‐K treated MEVs (pkt‐MEVs) and regular untreated MEVs to target M2 macrophages. MEVs were labeled with the lipophilic fluorescent dye, DiI, and further purified from free dye before incubation with M2 macrophages. Equal numbers of DiI‐labeled pkt‐MEVs and DiI‐labeled MEVs were left to incubate with separate M2 macrophage cultures for 0.5, 1, 1.5, and 2 h. We used wide‐field microscopy to compare the uptake of fluorescently labeled pkt‐MEVs by M2 BMDMs. We found that the proteinase‐K treatment of MEVs resulted in a 20% reduction in the uptake of MEVs by M2 macrophages (Figure 4b–d). Even after the digestion of membrane proteins present on MEVs, the limited loss of cellular uptake of pkt‐MEVs compared to untreated MEVs indicates that MEV uptake by M2 macrophages is not solely dependent on ligand–receptor interaction but likely driven by the inherent phagocytotic ability of M2 BMDMs.

We next tested the ability of proteinase K‐treated MEVs (pkt‐MEVs) to reprogram M2 macrophages toward an M1 phenotype. We incubated M2 macrophages with different concentrations of pkt‐MEVs and assessed the release of the pro‐inflammatory cytokine TNF‐*α* secreted by M2 macrophages compared to TNF‐*α* secreted by M2 macrophages that had been incubated with the corresponding concentration of undigested MEVs. We found that eliminating the proteins on the surface of MEVs resulted in a near‐complete loss in the ability of MEVs to re‐polarize M2 BMDMs toward an M1 phenotype (Figure 4e). This suggests that interactions between the membrane proteins present in MEVs, and the surface proteins present in M2 macrophages are the primary driver of MEV‐induced macrophage repolarization.

### 2.4 Programming Nanovesicles with Endogenous Ligands for Enhanced Macrophage Repolarization

**Figure 5:**
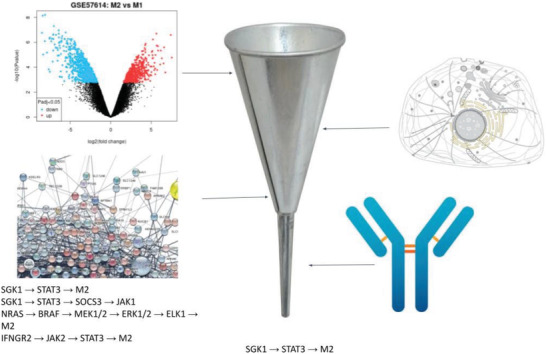
Experimental and computational workflow to identify MEV gene targets that promote M1 or disfavor M2. A list of localized proteins in the cell membrane and function as receptors was generated from the UniProt database. Pathways from the STRING database, KEGG, and our own curated database were generated to form a network of interconnected genes. Additionally, a list of proteins from an antibody experiment done on M1 was available. Cross‐referencing these lists, from differentially expressed genes, pm‐localized genes, network nodes, and antibody data, we can narrow down potential targets for M1 polarization.

In the previous section, we demonstrate that membrane proteins anchored on MEVs play a vital role in MEV‐mediated M2 to M1 polarization. To identify membrane proteins that might enhance macrophage polarization in response to MEV delivery we used a computational pathway analysis protocol we developed and reported previously^[^ ^24^ ^]^ (**Figure** **5**). For this, we first hypothesized that M1‐derived MEVs contain significantly higher expression levels of proteins that support or suppress pathways associated with M1 versus M2 polarization. To test our hypothesis, we used messenger ribonucleic acid (mRNA) data from National Center for Biotechnology Information (NCBI) GSE57614^[^ ^25^ ^]^ that we found to be consistent with the data in Figure 3 to first identify differentially expressed genes (DEGs) in M1 macrophages. We calculated logFC (defined as log(M1/M2)) in Table S1 (Supporting Information), where positive logFC indicates upregulation of genes for TNF*α*, IL6, and IL1*β* and downregulation of genes for CD36, ADORA3, and TGFBR2 in M1 macrophages. We found that the logFC values shown in Table S1 (Supporting Information) for transcripts IL6, IL1B, TNF‐*α*, CCL5, and ICAM1 indicated their upregulation whereas transcripts representative of anti‐inflammatory phenotype include TGFBR2,^[^ ^26^ ^]^ ADORA3,^[^ ^27^ ^]^ FFAR4^[^ ^28^ ^]^ are downregulated. LogFC values obtained from our computational approach were in fact representative of our experimental results. We observed that CD54 (ICAM1) is elevated in M1 and MEVs and CD9 is downregulated in M1 macrophages and MEVs relative to M2‐polarized macrophages (Figure 1c). In addition, our experimental results are consistent with the RNA data, as CD54 (ICAM1) is up‐regulated and CD9 is down‐regulated in M1 phenotypes. Additionally, CD206 (MRC1), a known M2 macrophage marker, is downregulated in the RNA data.

**Figure 6:**
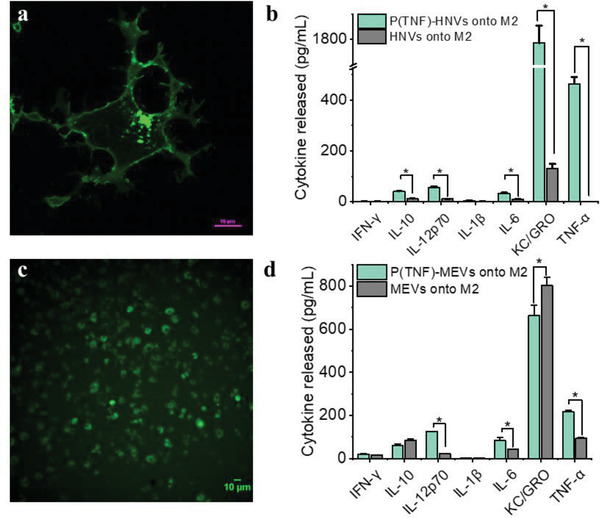
TNF‐*α* programmed nanovesicles cause M2 macrophages to repolarize more toward the M1 phenotype. a) Fluorescence image of HEK cells transfected with a mouse GFP‐tagged TNF‐*α* plasmid. Green fluorescence in the cell membrane indicated a clear overexpression of TNF‐*α* on the cell membrane. b) IFN‐*γ*, IL‐10, IL‐12p60, IL‐1*β*, IL‐6, KC/GRO, and TNF‐*α* released by M2 macrophages after incubation with TNF‐α programmed HEK‐cell derived nanovesicles (P(TNF)‐HNVs) and HNVs. c) A fluorescence image of macrophages transfected with a mouse GFP‐tagged TNF‐*α* plasmid. d) Comparison of IFN‐*γ*, IL‐10, IL‐12p60, IL‐1*β*, IL‐6, KC/GRO, and TNF‐*α* released by M2 macrophages incubated with P(TNF)‐MEVs and regular M1 macrophage‐derived vesicles (MEVs). Each data point is the average of at least 3 experiments (*n* = 3). The data is presented as the mean ± SEM. *p < 0.01 indicates that the results are statistically significant.

We next cross‐referenced the genes identified above against our manually curated database of macrophage signaling pathways. This database was represented by a network whose nodes corresponded to genes or expressed proteins, with interactions shown by directed edges. The edges were weighted to reflect a) whether the upstream node activates or inhibits the downstream node, b) if the node is substantiated by mRNA transcripts, and c) if the node is confirmed by mAb. We then conducted a search for paths in the network that agonized M1‐associated pro‐ versus anti‐inflammatory phenotypes for each gene localized to the plasma membrane. Candidate plasma‐membrane receptors and their corresponding ligands driving pro‐inflammatory pathways that were identified by our approach are listed in Table S2 (Supporting Information). The highest‐ranked pathways include the TNF and interleukin‐1 receptors, which activate pathways well‐known to polarize macrophages into pro‐inflammatory states.^[^ ^22^, ^29^ ^]^ Based on these results, we predicted TNF‐α as one of the possible targets for overexpression in MEVs aiming to achieve MEVs with the highest likelihood of polarizing target macrophages. To validate our strategy, we overexpressed TNF‐*α* on the cell membrane and developed TNF‐*α* overexpressing programmed to modulate macrophage polarization. In order to program cells by over‐expressing TNF‐*α* on their membrane, we first transfected HEK cells, then proceed to mouse bone marrow‐derived macrophages. We used a green fluroescent protein (GFP)‐tagged mouse TNF‐*α* plasmid for transfection to identify expression. TNF‐*α* expressing HEK cells were used to generate programmed HEK cell‐derived nanovesicles P(TNF)‐HNVs. We polarized TNF‐*α* expressing BMDMs to an M1 phenotype using LPS + IFN‐*γ* and used those cells for generating programmed macrophage‐engineered vesicles (P(TNF)‐MEVs). We confirmed TNF‐*α* overexpression on the cells using confocal microscopy which showed clear GFP fluorescence on the surface of these programmed cells (**Figure** **6**a,c). We then compared the macrophage repolarization efficacy of programmed nanovesicles including P(TNF)‐HNVs and P(TNF)‐MEVs relative to their respective controls, using equal concentrations of vesicles in the experimental and control groups. The repolarization efficacy of the programmed nanovesicles was evaluated based on the cytokine production of nanovesicle‐treated M2 BMDMs. We found that incubating M2 BMDMs with programmed HEK cell‐derived nanovesicles P(TNF)‐HNVs resulted in high levels of pro‐inflammatory cytokines such as IL‐12p70, IL‐6, IL‐10, KC/GRO, and TNF‐*α* (Figure 6b). However, unmodified HEK cell‐derived vesicles elicit virtually no proinflammatory cytokine production in M2 BMDMs. Consistent with our computational pathways analysis approach, we also found that M2 macrophages treated with programmed MEVs P(TNF)‐MEVs produce significantly higher levels of pro‐inflammatory cytokines such as Il‐12. These results indicate that, under similar conditions, programmed nanovesicles exhibit greater immunomodulatory properties compared to unmodified cell‐derived vesicles illustrating the capability to program functionality into MEVs via protein expression. Our computational pathway analysis approach indicated that the interaction of TNFSR plasma membrane receptors with TNF‐*α* could activate an NF‐kB signaling pro‐inflammatory pathway that shifts macrophages into pro‐inflammatory states. Previous studies have also implicated the activation of the nuclear factor kappa B (NF‐kB) pathway by TNF‐*α* in macrophages.^[^ ^22a^ ^]^ These studies suggest that NF‐kB may be one of the possible signaling pathways driving MEV‐mediated M2 to M1 repolarization.^[^ ^30^ ^]^ Nuclear factor‐kB (NF‐kB) is one of the main transcription factors of M1 macrophages and regulates the expression of genes that control factors such as inflammation.^[^ ^31^ ^]^ Activation of NF‐kB is characterized by the nuclear translocation of the p65 component of the NF‐kB complex.^[^ ^32^ ^]^ To determine if MEV delivery to M2 BMDMs activated the NF‐kB signaling pathway, we incubated M2 Macrophages with MEVs for 6 h, then fractionated the cells into cytoplasmic and nuclear fractions to study the effects of MEV delivery on the translocation of p65 subunits to the nucleus. We performed western blotting analysis to compare the p65 content in MEV‐treated M2 BMDMs using untreated M1 and M2‐BMDMs as controls. Western blotting analysis indicated a significant translocation of p65 from the cytoplasm to the nucleus indicating the NF‐kB pathway is activated when MEVs interact with M2 BMDMs (Figure S3, Supporting Information).

**Figure 7:**
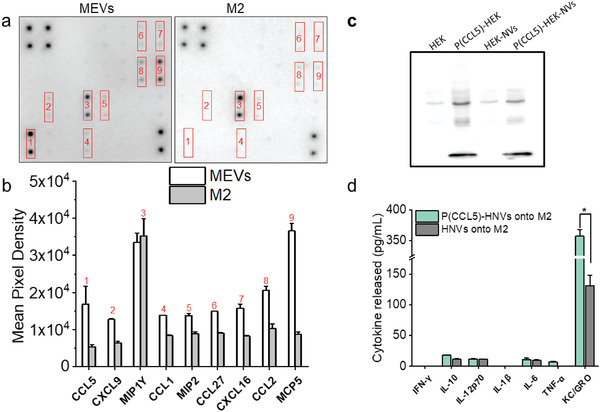
CCL5‐programmed nanovesicles for M2 to M1 macrophage reprogramming. a) Chemokine expression image of the chemokine antibody array for MEVs and M2 macrophages. Dark spots in the images indicate the presence of the specific chemokine. b) Comparison of mean pixel integrated density measurements between chemokines present on MEVs and M2 macrophages. c) Western blotting shows that programmed HEK cells express more CCL5 than regular HEK cells. Accordingly, vesicles generated from programmed HEK cells express a greater CCL5 concentration. d) CCL5‐programmed vesicles derived from HEK stimulate M2 BMDMs to produce more KC/GRO. Each data point is the average of at least 3 experiments (*n* = 3). The data is presented as the mean ± SEM. *p < 0.01 indicates that the results are statistically significant.

We next sought to improve the capability of nanovesicles to modulate macrophage polarization by generating programmed nanovesicles that overexpress specific membrane‐bound chemokines. Chemokines are chemotactic cytokines produced by various cells including macrophages.^[^ ^33^ ^]^ While chemokines are mostly known for their role in monocyte recruitment/migration, they can promote macrophage differentiation as well as polarization.^[^ ^10^, ^34^ ^]^ For example, C–C motif chemokine ligand 5 (CCL5) has been shown to activate M1 polarization and inhibit M2 polarization.^[^ ^35^ ^]^ Based on our computational pathway analysis approach results in Table S2 (Supporting Information), we found that C‐C motif chemokine receptor 3 (CCR3) and CCR5 plasma membrane receptors drive pro‐inflammatory pathways and are among the highest‐ranked pathways that polarize macrophages into pro‐inflammatory states. Therefore, we first analyzed MEVs for the presence of 25 different macrophage‐associated chemokines that could interact with CCR3 or CCR5 and modulate M2 macrophages toward the M1 phenotype. We found that only nine chemokines including C–C motif chemokine ligand 5 (CCL5), C–X–C motif chemokine ligand 9 (CXCL9), macrophage inflammatory protein 1‐*γ* (MIP1‐*γ*), C–C motif chemokine ligand 1 (CCL1), macrophage inflammatory protein‐2 (MIP‐2), C–C motif chemokine ligand 27 (CCL27), C–X–C motif‐ chemokine ligand 16 (CXCL16), C–C motif chemokine ligand 2 (CCL2), and monocyte chemotactic protein 5 (MCP‐5) were found to be present on MEVs (**Figure** **7**a). We next compared the level of these observed chemokines for MEVs compared to M2 macrophages. We found that several M1‐polarizing chemokines including CCL2,^[^ ^34^, ^36^ ^]^ CCL5,^[^ ^35^ ^]^ and CXCL9^[^ ^37^ ^]^ were present at significantly higher levels in MEVs compared to M2 macrophages (Figure 7a,b). We next evaluated the CCR3 pathway. CCR3 is activated by several ligands, including CCL5.^[^ ^38^ ^]^ Treatment of peritoneal macrophages and bone‐derived macrophages with CCL5 has been shown to activate MAPK and NF‐kB, which are associated with the M1 proinflammatory state.^[^ ^35^ ^]^ Namely, CCL5 was shown to produce proinflammatory cytokines such as TNF‐*α* via the NF‐kB pathway through the phosphatidylinositol 3‐kinase (PI3K)/protein kinase B (AKT) pathway. However, CCL5 has also been reported to have a higher affinity to CCR5 than CCR3.^[^ ^39^ ^]^ Overexpressing CCL5 can therefore potentially preferentially activate CCR5, which is associated with polarization to an anti‐inflammatory state.^[^ ^40^ ^]^ This may occur via the MEK/STAT3 pathway.^[^ ^40b^ ^]^ To reconcile these disparate outcomes, we proposed that over‐expression of CCL5 would acutely activate the CCR3 pathway and thereby drive pro‐inflammatory responses in MEV‐treated TAMs. To test this concept, we next generated programmed CCL‐5 overexpressing HEK cell‐derived nanovesicles (P(CCL5)‐HNVs) and compared the macrophage repolarization efficacy of (P(CCL5)‐HNVs) with unmodified HEK cell‐derived nanovesicles. HEK nanovesicles themselves do not polarize M2 macrophages. Therefore, by overexpressing CCL5 on HEK nanovesicles, we can determine the role of CCL5 present in macrophage modulation. We confirmed CCL5 expression in programmed HEK cells as well as programmed nanovesicles by western blotting (Figure 7c). While HEK cells did not express mouse CCL5 prior to transfection, programmed HEK cells and programmed nanovesicles showed a clear band for CCL5, confirming CCL5 expression in both programmed cells and programmed nanovesicles. In order to compare the repolarization efficacy of programmed vesicles relative to regular HEK vesicles, we added 4 × 10^9^ vesicles to 50 000 M2 macrophages in culture, incubated for 24 h and tested the cell culture supernatants for pro‐inflammatory cytokines. We found that M2 macrophages that were left to incubate with CCL5‐programmed HEK cell‐derived nanovesicles (P(CCL5)‐HNVs) produced roughly three times more KC/GRO compared to M2 macrophages that were incubated with regular HEK cell‐derived nanovesicles (Figure 7d). However, we did not observe significant differences in the production of other cytokines by M2 macrophages. We found comparable results when we incubated M2 BMDMs with CCL5‐programmed nanovesicles generated using M1 macrophages (Figure S4, Supporting Information). These results indicate that, under similar in vitro conditions, CCL‐5 programmed nanovesicles can cause higher keratinocyte chemoattractant (KC)/human growth regulated oncogene (GRO) production by M2 macrophages. However, CCL5‐programmed nanovesicles do not increase other pro‐inflammatory cytokine production by M2 BMDMs.

**Figure 8:**
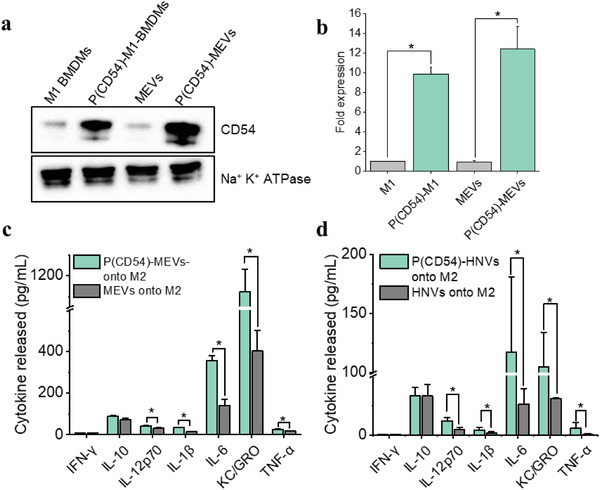
ICAM‐1 programmed nanovesicles mediated M2 to M1 macrophage reprogramming. a) Western blotting analysis reveals that CD54 transfected programmed M1 macrophages (P(CD54)‐M1) express more CD54 than non‐transfected M1 macrophages. As a result, vesicles derived from programmed macrophages (P(CD54)‐MEVs) have a higher concentration of ICAM‐1. b) A bar graph displaying the relative fold of CD54 expression in M1 macrophages, programmed M1 macrophages, MEVs, and P(CD54)‐MEVs. CD54 expressions are normalized to Na^+^K^+^ ATPase, a plasma membrane marker. c,d) CD54‐programmed vesicles exhibit higher repolarization efficiency compared to regular nanovesicles. Each data point is the average of at least 3 experiments (*n* = 3). The data is presented as the mean ± SEM. *p < 0.01 indicates that the results are statistically significant.

We have established that MEVs express various exosomal marker proteins, including CD54 (ICAM‐1), CD63, MHCII, CD11b, and CD81 (Figure 1c). Recent studies have shown that ICAM‐1 prevents M2 polarization and inhibits tumor metastasis.^[^ ^21a^ ^]^ ICAM1 is a cell surface glycoprotein produced in breast cancer cells following their exposure to pro‐inflammatory cytokines.^[^ ^41^ ^]^ The pathway we identified suggests that ICAM1 produces TNF‐*α* and IFN‐*γ* via the MAPK signaling pathway after binding and activating receptors ITGAL (integrin subunit alpha L) and ITGB2.^[^ ^42^ ^]^ ICAM1 also supports the expression of IL8, CCL3, and CCL4 by prolonging the stability of TNF mRNA.^[^ ^43^ ^]^ ICAM1 was also shown to inhibit M2 polarization^[^ ^21a^ ^]^ by suppressing PTEN, which is a negative regulator of PI3K/AKT. We, therefore, suggested that overexpression of pro‐inflammatory ligands induced by cytokines may constitute another strategy for priming M1 phenotypes in TAMs. Therefore, we generated programmed nanovesicles that overexpress CD54 to assess their efficacy in macrophage phenotype modulation. For this, we first transfected M1 macrophages with a mammalian expressing mouse ICAM‐1 plasmid, then polarized them to an M1 phenotype and used those macrophages to generate programmed macrophage‐engineered vesicles (P(CD54)‐MEVs). We performed western blotting analysis to obtain the relative expression level of ICAM1 both in programmed macrophages as well as programmed MEVs relative to control. Western blotting analysis demonstrates that programmed cells and programmed nanovesicles express about tenfold higher concentrations of ICAM1 relative to their respective controls (**Figure** **8**a,b). We next compared the macrophage repolarization efficacy of (P(CD54)‐MEVs) with regular MEVs by delivering an increasing concentration of each type of MEV onto M2 macrophages and quantifying the cytokine released by MEV‐treated M2 BMDMS. We found that M2 BMDMs incubated with programmed MEVs (P(CD54)‐MEVs) produced higher levels of pro‐inflammatory cytokines compared to M2 BMDMs incubated with the same number of unmodified MEVs (Figure S5, Supporting Information). When we analyzed the cytokine release by M2 BMDMs incubated with the specific concentration of MEVs (4 × 10^9^), we found that M2 macrophages incubated with programmed MEVs produced roughly three times more KC/GRO, two times more IL‐6 and IL‐1*β*, and 25% more IL‐12p70 and TNF‐*α* compared to M2 macrophages that were incubated with non‐programmed MEVs (Figure 8c). As a control study, we generated CD54‐overexpressing programmed nanovesicles using CD54‐transfected HEK cells and then delivered these nanovesicles to M2 BMDMs in culture. We found similar results when we incubated M2 BMDMs with vesicles derived from HEK cells (Figure 8d). Our computational pathways analysis approach predicted that the ICAM1‐mediated pathway could upregulate the expression of IL‐1*β*, Il‐6, and TNF‐*α* in MEV‐treated M2 macrophages. Our experimental results indicate consistent increases in most pro‐inflammatory cytokines as predicted by our computational strategy. These results further support the idea that endogenous proteins can be overexpressed in programmed nanovesicles to yield greater immunomodulatory properties compared to unmodified cell‐derived vesicles.

### 2.5 Programming MEVs with Exogenous Ligands for Enhanced Macrophage Modulation

**Figure 9:**
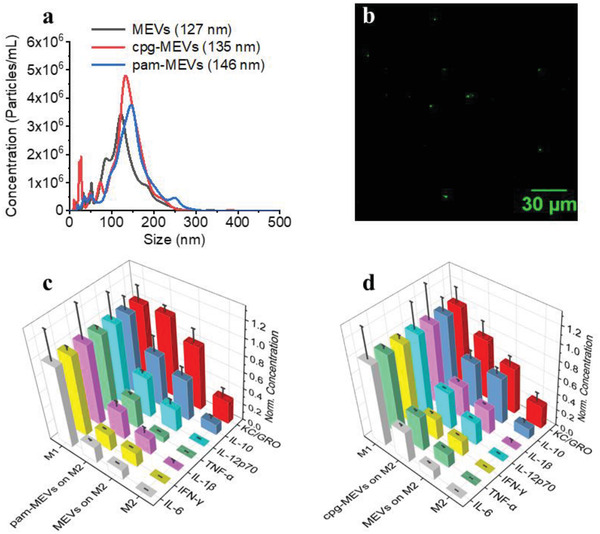
Programming MEVs with TLR‐ligands for enhanced M2 macrophage reprogramming toward an M1 phenotype. a) Size distribution graph for MEVs, CpG oligonucleotide incorporated MEVs (cpg‐MEVs), and Pam3CSK4 decorated MEVs (pam‐MEVs) from Nanoparticle Tracking Analysis. The mean diameter of MEVs (black), cpg‐MEVs (red), and pam‐MEVs (blue) is 127, 135, and 146 nm, respectively. b) Fluorescence image of rhodamine‐labeled Pam3CSK4 decorated MEVs demonstrating successful MEV programming via Pam3CSK4 decoration. c) Comparison of the repolarization efficiency of Pam3CSK4 ligand decorated MEVs compared to regular MEVs. d) Comparison of the repolarization efficiency of cpg‐ODN ligand incorporated MEVs compared to regular MEVs. Each data point is the average of at least 3 experiments (*n* = 3). The data is presented as the mean ± SEM.

The incorporation of specific non‐endogenous ligands into MEVs could yield improved levels of M2 macrophage reprogramming capability as these ligands have the potential to directly interact with receptors present on M2 macrophages and initiate downstream signaling cascades. Here, we identified pathways associated with TLR1 and TLR2 activation, which are toll‐like receptors that dimerize upon the binding of lipopeptides.^[^ ^44^ ^]^ Activation of these toll‐like receptors in turn primes pro‐inflammatory pathways mediated by myeloid differentiation primary response 88 (MyD88).^[^ ^45^ ^]^ The TLR1/2/4‐NF‐kB signaling pathway we identified, using a pathway analysis approach, shows the production of pro‐inflammatory cytokines TNF‐*α*, IL1B, IL6, and IL12 in two ways: PI3K‐AKT signaling pathway (RAC1‐PI3K‐AKT‐NF‐kB) and mitogen‐activated protein kinases (MAPK) signaling pathway.^[^ ^42^ ^]^ However, it also shows the production of pro‐inflammatory cytokines via NF‐kappa‐B inhibitor alpha.^[^ ^42^, ^46^ ^]^ We, therefore, proposed TLR1/2 stimulation via compounds like N‐Palmitoyl‐S‐[2,3‐bis(palmitoyloxy)‐(2RS)propyl]‐[R]‐cysteinyl‐[S]‐seryl‐[S]‐lysyl‐[S]‐lysyl‐[S]‐lysyl‐[S]‐lysine (Pam3CSK4) to determine the capacity of these receptors’ activation in priming M1 polarization. To validate our strategy, we first generated macrophage‐engineered vesicles from mouse pro‐inflammatory M1 macrophages (MEVs) and then Pam3CSK4, a toll‐like receptor 2(TLR2)/TLR1 ligand, was grafted onto the lipid bilayer membrane of the vesicles by sonication. We used Rhodamine‐labeled Pam3CSK4 to confirm the successful decoration of Pam3CSK4 on the surface of vesicles. Green punctate regions in **Figure** **9**b, a widefield fluorescence image of MEVs that had been programmed with Rhodamine‐labeled Pam3CSK4, showed the successful decoration of Pam3CSK4 on the surface of MEVs. We used nanoparticle tracking analysis to compare the size of these vesicles and found that Pam3CSK4 grafted vesicles (pam‐MEVs) were slightly larger (146 nm) as compared to MEVs (127 nm) (Figure 9a). We found that the zeta potential of pam‐MEVs was −16.8 mV indicating improved stability in aqueous solution. To compare the capability of MEVs and pam‐MEVs to reprogram M2 macrophages, we incubated M2 BMDMs separately with an equal number of MEVs or pam‐MEVs and compared their cytokine production. While M2 macrophages show no detectable proinflammatory cytokines, those incubated with pam‐MEVs showed substantial levels across most cytokines. Comparing cytokine levels we typically observe for M1 polarized macrophages, we saw that pam‐MEV treatment shifted M2 macrophages to 20 ± 2% (IFN‐*γ*), 64 ± 20% (IL‐10), 50 ± 4% (IL‐12p70), 35 ± 7% (IL‐1β), 20 ± 2% (IL‐6), 100 ± 7% (KC/GRO) and 36 ± 3% (TNF‐*α*) of the average concentration seen for M1 macrophages (Figure 9c). Similarly, M2 macrophages incubated with standard MEVs, exhibited values of 17 ± 1% (IFN‐*γ*), 51 ± 11% (IL‐10), 28 ± 6% (IL‐12p70), 20 ± 8% (IL‐1*β*), 12 ± 1% (IL‐6), 79 ± 16% (KC/GRO) and 7 ± 1% (TNF‐*α*) of the average concentration seen for M1 macrophages (Figure 9c). We found that pam‐MEVs treated M2 macrophages were more efficient at reprogramming M2 macrophages toward a proinflammatory phenotype than unmodified MEVs.

We also generated programmed nanovesicles by incorporating small molecule agonists on the vesicle surface. Cytosine‐phosphorothioate‐guanine oligodeoxynucleotides (CpG‐ODN), a synthetic oligonucleotide, has sequence patterns that resemble bacterial DNA and has been found to activate APCs such as macrophages and dendritic cells.^[^ ^47^ ^]^ CpG‐ODN is recognized by the Toll‐like receptor 9 (TLR‐9) present in APCs.^[^ ^48^ ^]^ CpG‐ODN interaction with TLR‐9 initiates signaling pathways that causes the APC to secrete several pro‐inflammatory cytokines including IFN‐*γ*, IL‐12p70, and TNF‐*α*.^[^ ^48a^ ^]^ Recent studies have shown that stimulation of macrophages with ODN1826, a Class B CpG oligonucleotide, increases their phagocytotic and anti‐tumor activity.^[^ ^13^ ^]^ CpG‐ODN‐treated macrophages produced high levels of pro‐inflammatory cytokines compared to other immune‐stimulant agonists upon interaction with macrophages.^[^ ^13^ ^]^ While CpG‐ODN is a well‐known immunostimulatory agonist, its efficacy is limited by its rapid degradation and inability to effectively be delivered to the intracellular compartment of APCs.^[^ ^49^ ^]^ We incorporated ODN1826 into the membrane bilayer of MEVs. To compare the capability of MEVs and cpg‐MEVs to reprogram already polarized macrophages, we separately incubated M2 BMDMs with an equal number of MEVs or cpg‐MEVs and compared cytokine production. Cpg‐MEVs induced the production of cytokines to levels of 26 ± 3% (IFN‐*γ*), 58 ± 11% (IL‐10), 46 ± 1% (IL‐12p70), 41 ± 3% (IL‐1*β*), 38 ± 3% (IL‐6), 75 ± 18% (KC/GRO), and 43 ± 2% (TNF‐*α*) of the average concentration seen for M1 macrophages (Figure 9d). This was an improvement over the polarization induced by unmodified MEVS of 7%, 1%, 18%, 10%, 26%, 18%, and 26% higher efficiency in the production of IFN‐*γ*, IL‐10, IL‐12p70, IL‐1*β*, IL‐6, KC/GRO and TNF‐*α*, respectively. These results suggest MEV efficacy can be enhanced by incorporating polarization‐inducing ligands into the surface of the vesicle.

## 3 Conclusion

In conclusion, exosome‐mimicking vesicles can be generated from BMDMs with high yield using nitrogen cavitation. Interactions of endogenous ligands present on the membrane bilayer of a vesicle with their corresponding receptors present on the target macrophage cause anti‐inflammatory macrophages to repolarize toward a pro‐inflammatory phenotype. Cell‐engineered nanovesicles can be programmed to overexpress specific ligands on their surface that improve targeting and repolarization efficacy. Programmed nanovesicles, when interacting with the M2 macrophages can elicit enhanced immunomodulatory properties compared to non‐programmed vesicles. Programmed vesicles exhibited negative zeta potentials (Table S3, Supporting Information) indicating long‐term stability in solution. This shows that programmed M1 macrophage‐engineered nanovesicles have the potential to be used as a therapeutic platform to achieve enhanced re‐polarization of tumor‐supportive M2 macrophages toward a tumor‐killing M1 phenotype. Macrophage polarization exists in a continuum ranging across a spectrum from proinflammatory to anti‐inflammatory and is most often characterized by cytokine release. While we have demonstrated that several strategies can be used to enhance the ability of MEVs to repolarize macrophages toward a proinflammatory phenotype, the extent and the profile of the proinflammatory properties (e.g., cytokine release) vary across the different ligands and proteins featured on the surface of the vesicle. The specific cytokines increased by the ligands are likely reflective of the signaling pathways initiated through interactions with the target macrophage. The optimal strategy for programming MEVs for their ability later macrophage phenotype may require a mixture of surface functional groups to enhance the overall proinflammatory properties or may require a tailored approach that utilizes ligands that specifically elicit the production of one or more cytokines. This work does establish that surface proteins on MEVs are responsible for their inherent immunomodulatory properties. By introducing specific characteristics on the surface of the vesicle's membrane, we can adjust and direct the process of repolarization toward either a pro‐inflammatory or anti‐inflammatory state. The capability to shift macrophages between polarization phenotypes offers a promising method that could lead to alternative treatments for several diseases. For instance, redirecting tumor‐associated macrophages to a pro‐inflammatory state holds promise as a strategy to increase the effectiveness of immunotherapy in treating cancer. Similarly, transforming pro‐inflammatory macrophages into anti‐inflammatory phenotypes could be a favorable approach to mitigate the potential neurotoxic effects associated with M1 macrophages, offering potential treatments for a wide range of diseases related to inflammation as well as conditions such as spinal cord injury. Additionally, the technique for generating vesicles and subsequently functionalizing their surface features is readily scalable to large‐scale production. The studies here utilized a relatively small volume of cells (10 mL for ≈100 million cells). Similar nitrogen cavitation vessels with 10 to 100 times the capacity are readily available. Our approach should be readily scalable to production levels.

## 4 Experimental Section

### Animals

Two to five‐month‐old C57/BL6 mice were used to harvest bone marrow cells. Mice that were initially purchased from the Jackson Laboratory and were appropriately housed in IVC with a sufficient supply of food and water. All experiments were conducted in accordance with National Institutes of Health guidelines that were authorized by the University of Kentucky's Institutional Animal Care and Use Committee (IACUC: Kolesar 2017–2674).

### Cell Culture

Mouse bone marrow‐derived macrophages (BMDMs) were harvested from two to five‐month‐old mice following previously published methods.^[^ ^14^ ^]^ Bone marrow from the femurs and tibias of mice was first flushed and then homogenized using Dulbecco's Modified Eagle's Medium (DMEM) media. The solution of cells was then centrifuged at 1000 × *g* for 5 min to collect the cell pellet. Red blood cell (RBC)‐lysis buffer containing 0.15 m NH4Cl, 10 mm KHCO3, and 0.1 mm Na4EDTA was then used to lyse the erythrocytes present in the cell pellet. Erythrocyte‐free monocytes were then cultured in a 75‐cm^2^ culture flask containing 12 mL of cell culture medium. Mouse bone marrow‐derived macrophage cell culture medium contained RPMI media supplemented with 20% cell‐culture supernatant obtained from sL929 cells, 10% fetal bovine serum (FBS), 5% penicillin/streptomycin, 1% glutamine, 1% HEPES, and 0.001% 2‐mercaptoethanol. The cell culture medium was changed every two days. On day 7, cells were re‐seeded at a cell density of 1 × 10^6^ cells mL^−1^ in Dulbecco's modified eagle medium (DMEM) media supplemented with 10% FBS, 1% glutamine, and 1% penicillin/streptomycin and stimulated to M1‐macrophages using LPS (20 ng mL^−1^) and IFN‐*γ* (20 ng mL^−1^) or to M2‐macrophages using IL‐4 (20 ng mL^−1^).

### Vesicles Isolation

Previously published methods^[^ ^9^, ^14^ ^]^ were followed to generate pro‐inflammatory MEVs. Briefly, to generate MEVs, used fully differentiated pro‐inflammatory (M1) macrophages were used. First, cells were rinsed with PBS and then resuspended in PBS containing one protease inhibitor tablet per 10 ml of 1× PBS buffer. The cell slurry was then transferred to a nitrogen cavitation vessel on ice and subjected to 300 psi of nitrogen gas pressure for 5 min. The rapid release of pressure caused the fragmentation of cells and the subsequent formation of vesicles. The nitrogen cavitation‐obtained cell lysate was centrifuged at 4000 × *g* for 10 min at 4 °C. The pellet obtained after centrifugation was discarded, and the supernatant was centrifuged again for 20 min at 10 000 × *g* and 4 °C. The obtained supernatant was ultracentrifuged at 100 000 × *g* for 60 min at 4 °C to obtain a pellet containing MEVs. The pellet was then resuspended in phosphate buffered saline (PBS) to release MEVs in the solution.

### Nanoparticle Tracking Analysis

Nanoparticle tracking analysis (NTA) was used to determine the size and concentration of MEVs in samples using a NanoSight NS300 equipped with NTA analytical software and a 488 nm laser. Samples of MEVs were diluted one thousand times with ultrapure water, and five 30‐second videos were recorded for analysis. The parameters for the analysis software remained constant for all measurements of any given sample.

### Transmission Electron Microscopy Imaging of MEVs

Vesicles were resuspended in PBS and fixed in 2% glutaraldehyde. Copper TEM grids with an ultra‐thin carbon layer and lacey film (Ted Pella, Inc) were glow discharged at 10 mA for 15 s. Vesicles were deposited on the grids for 1 min. The excess solution was removed, and vesicles were negatively stained with 2% uranyl acetate for 20 s. Excess uranyl acetate was removed, and grids were air‐dried. Vesicle micrographs were collected using a Talos F200X TEM (Thermo Scientific) operating at 200 kV accelerating voltage. The contrast was enhanced by inserting a 50 µm objective aperture.

### Western Blotting

Vesicle pellets and cells were initially treated in the radioimmunoprecipitation assay (RIPA) buffer (150 mm NaCl; 1% Triton X‐100; 0.5% sodium deoxycholate; 0.1% SDS; 50 mm Tris pH 8.0; 1x protease inhibitor cocktail (Roche)). Each protein sample (50 µg) was resolved on 12% polyacrylamide gels and then transferred to a nitrocellulose membrane. For comparative experiments, an equal protein from various samples was loaded per lane. The membrane was then blocked for 1 hour with 5% milk in TBST and incubated with primary antibodies at room temperature for 2 h with constant shaking. After washing and removal of the primary antibody, the HRP‐conjugated secondary antibody was added for 1 h. The membranes were washed with TBST and the protein bands were developed using chemiluminescent detection.

### Cytokine Quantification

Macrophages were seeded at a density of 50 000 in 96 well plates and then stimulated to M1 or M2 phenotypes as discussed in the previous section. For the cytokine analysis, macrophage conditioned medium (MCM) was collected after 24 hours of stimulation or treatment with MEVs. The MCM was frozen at −80 °C until analysis. Following the manufacturer's instructions from Meso Scale Discovery (MSD), a mouse seven‐plex pro‐inflammatory cytokine test was conducted to simultaneously detect IFN‐*γ*, IL‐10, IL‐12p70, IL‐1*β*, IL‐6, KC/GRO, and TNF‐*α* present in the MCM. Calibrators (50 µL) and MCM were used for cytokine analysis. MESO SECTOR imager from Meso Scale Delivery was used to analyze the MSD plate for pro‐inflammatory cytokines. All samples were run in triplicates and used vesicle suspensions in PBS to measure the proinflammatory cytokines present in the vesicle suspension. In some experiments, the same protocol as previously discussed was used to measure the concentration of TNF‐*α* in the MCM using a single spot well plate.

### Chemokine Quantification

Chemokines present on the MEVs were detected using Mouse Chemokine Array C1 (Ray Biotech, Code AAM‐CHE‐1‐2) and semi‐quantified following the manufacturer's protocol. Briefly, chemokines were first extracted from MEVs and M2 macrophages, a control, using cell lysis buffer as provided by the manufacturer. The protein concentration in M1EVs and M2 samples was measured using a UV/VIS spectrophotometer. For proteomic analysis, first, the antibody arrays were incubated in 2 mL of blocking buffer for half an hour with constant shaking at room temperature. After half an hour, the blocking buffer was aspirated off and ≈500 µg of the protein samples were loaded into each well of an incubation array and left to incubate for 3 h at room temperature. Following several washes, a biotinylated antibody cocktail was added to each well and incubated for 2 hours at room temperature. The biotinylated antibody cocktail was then aspirated off, followed by multiple washings. After this, 1× horseradish peroxidase (HRP)‐Streptavidin was added to each well and incubated for 2 h at room temperature. After multiple washings, 500 µL of detection buffer mixture was added to each membrane and visualized by chemiluminescent detection. The immunoblot images were analyzed using ImageJ software.

### Coding Information

The code for this study is provided as a bitbucket repository that is currently available at bitbucket.org:pkhlab/pathwayanalysis.git. A website for its usage and an example ipython notebook is provided at https://bending456.github.io/Macrophage/ and https://colab.research.google.com/drive/12jtuAzQAvVtMoYm2EhInrxKgyit99Vc9?usp=sharing

### mRNA Analyses

To find genes that were highly expressed or repressed in M1 macrophages, the authors searched for mRNA data that was publicly available on the NCBI GEO website. The mRNA data was retrieved from the gene expression microarray experiment in NCBI with GEO accession number GSE57614, PMID: 25 799 240^[^ ^25^ ^]^ The study included Transcriptomic analysis of human polarized macrophages, under four conditions: Resting macrophage (M0), M1 (stimulated using IFN‐*γ*, and LPS), M2a (by IL‐4) and M2c (by IL‐10). Transcription profiles were taken after 3‐time points: 6, 12, and 24 h, each with 3 replicates. M2a samples induced by IL‐4 were chosen over M2c induced by IL‐10. Macrophages were also stimulated for 24 h, so the use of the 24‐hour dataset instead of the 6‐ and 12 h was chosen to stay as close to the experiment as possible. The online statistical tool GEO2R was used to examine the raw gene expression data, which utilized R/Bioconductor and Limma package v3.26.8. Inbuilt statistical methods such as the *t*‐test and Benjamini and Hochberg (false discovery rate) were used to determine the DEGs. Two comparisons were made: 1) M0 versus M1 and 2) M2 versus M1. From these two lists of DEGs, genes with an adjusted *p*‐value less than 0.05 were considered significant. The two lists were then processed to find common genes found in both lists. This list was used to cross‐reference proteins localized in the plasma membrane.

### Plasma Membrane Localization

To find proteins localized to the plasma membrane, the authors turned to UniProt^[^ ^50^ ^]^ to look for annotations of proteins in the plasma membrane. Specifically, the authors searched for a location as “cell membrane” and function as “receptor”.  Annotations for plasma membrane proteins were downloaded, filtered, and processed.

### Network Analysis

A graph‐theoretic approach was adopted to evaluate macrophage signaling pathways. The signaling networks comprised nodes representing intracellular or plasma membrane proteins, while edges linking those proteins symbolized protein–protein interactions or communication via second messengers see middle panel Figure S6 (Supporting Information). The advantage of this representation is that there were well‐established algorithms for identifying optimal pathways that link two arbitrary nodes, such as a path that links receptor activation to a cytokine response typical of M1 macrophages. This approach was summarized in the upper‐right corner of Figure S6 (Supporting Information). The key steps to the approach were 1) the duration of signaling networks from databases and 2) pathway searching via network analysis algorithms. For 1), candidate pathways implicated in macrophages from databases that include the Kyoto Encyclopedia of Genes and Genomes (KEGG) and Wikipathways were manually collected. Cytoscape was used to merge and curate pathways into a broad network of protein–protein interactions. The resulting network topology was exported for analysis using NetworkX. NetworkX was a comprehensive Python library for performing routine graph analyses, including the identification of ’minimum first paths’ that linked a given receptor to an M1‐associated cytokine gene product. RNAseq and immunohistochemistry data reported in the literature or by our collaborators to align the curated network topologies with properties specific to a given macrophage subtype were additionally incorporated. Altogether, 1) and 2) yielded sets of pathways and the receptors that activated them in a rank‐ordered list. Additional details are summarized in Section 1 (Supporting Information) and online documentation is provided.

### STRING Network Extension

Using the STRING database extension software, edges were added to refine the network.^[^ ^51^ ^]^ This resulted in the pathways used for the search protocol. To infer whether an edge was agonistic versus antagonistic potential, it was assumed that if two genes had positively correlated changes in mRNA expression, the edge was agonistic; otherwise, a negative correlation was assumed to be antagonistic. However, most were deemed insignificant.

### Ligand Incorporation into MEVs

MEVs were generated from 150 million M1 macrophages and resuspended in a 500 µL solution containing ligands (Pam3CSK4, rhodamine‐labeled Pam3CSK4, or CpG‐ODN) at 1 mg mL^−1^. The ligand‐MEV solution was then sonicated using a Q125 sonicator with a 0.125″ tip with the following settings: 20% amplitude, 20 cycles of 30 s on/off for 10 min. The MEV‐ligand solution was allowed to cool down on the ice for two minutes between each cycle. After completion of the sonication cycle, the ligand‐MEV solution was left to incubate on ice for 60 min to allow the recovery of the MEV membrane. The ligand‐decorated MEVs containing solution was diluted to 4 mL in PBS and subjected to ultracentrifugation at 100 000 × *g* for 60 min at 4 °C to collect the pellet containing ligand‐decorated MEVs, which was washed with 1 mL of PBS twice and resuspended in 500 µL of regular macrophage media. The number of MEVs was determined using NTA. 1 × 10^11^ MEVs were then added into each well of a 96‐well plate containing 50 000 M2 macrophages in 100 µL of replating media. The plate was left to incubate for 24 h at 37 °C. After 24 h, MCMs were collected and used for pro‐inflammatory cytokine analysis.

### RelA Translocation Assay

The activation of the NF‐kB pathway by MEV was measured by comparing the quantity of the p65 subunit (RelA) that was translocated into the nucleus using a nuclear translocation assay. The NF‐kB Assay Kit (FIVE photon Biochemicals, San Diego, CA) was used to fractionate M1 macrophages, M2 macrophages, and M2 macrophages treated with MEV into nuclear and cytoplasmic fractions. The amount of p65 in both the nuclear and cytoplasmic fractions was determined by western blotting.

### Confocal Imaging

A Nikon A1R confocal microscope was used for confocal imaging of the cells that was programmed to overexpress specific ligands on their surface. Images were analyzed using Nikon Elements image processing software.

### Transfection

HEK cells were transfected using Lipofectamine 2000 reagent (Invitrogen) using the manufacturer's protocol. The authors used 2–5‐month‐old wild‐type C57BL/6 mice to isolate bone marrow monocytes. Monocytes were then differentiated into macrophages. On day five, macrophages were transfected with a plasmid. Macrophages were transfected using the jet PEI‐Macrophage in vitro DNA transfection reagent following the manufacturers protocol. Transfection efficiency was compared by confocal imaging and western blotting.

### Statistical Analysis

Statistical analyses were carried out using Origin. Data were reported as the mean ± standard deviation of the mean (SEM). At least three separate experiments were conducted for each condition (*n* = 3). A one‐way ANOVA was done to determine statistical significance with Tukey's post hoc analysis. The results were considered statistically significant if the *p*‐value was less than or equal to 0.01.

## Author Contributions

K.R.N., G.R.S., and C.I.R. wrote the manuscript. K.R.N., B.H., J.L., S.P.A., and M.A. performed the experiments. G.R.S. and B.C. performed computational pathway analysis. C.I.R., J.K., and J.C.G. designed the experiments.

## Conflict of Interest

The authors declare no conflict of interest.

## Supporting information

Supporting Information

## Data Availability

The data that support the findings of this study are available from the corresponding author upon reasonable request.
