# Supplementary material for: Programming Cell-Derived Vesicles with Enhanced Immunomodulatory Properties
Source: Adv Healthc Mater. Author manuscript; Available in PMC 2024 Oct 1. (PMC11070110; doi:10.1002/adhm.202301163)
Supplement: supp [file NIHMS1985395-supplement-supp.pdf]

ADVANCED HEALTHCARE MATERIALS

Supporting Information

for *Adv. Healthcare Mater.*, DOI 10.1002/adhm.202301163

Programming Cell-Derived Vesicles with Enhanced Immunomodulatory Properties

*Khaga R. Neupane, Geraldine S. Ramon, Brock Harvey, Byeong Chun, Surya P. Aryal, Abdullah A. Masud, J. Robert McCorkle, Jill M. Kolesar, Peter M. Kekenes-Huskey and Christopher I. Richards**

Supplementary Information

Programming Cell-Derived Vesicles for Enhanced Immunomodulatory Properties.

*Khaga R. Neupane†, Geraldine San Ramon¶, Byeong Chun¶, Brock Harvey†, Surya P. Aryal†, Abdullah A. Masud‡, Peter M. Kekenes-Huskey¶, Jill M. Kolesar‡, and Christopher I. Richards†**

†. Department of Chemistry, University of Kentucky, Lexington, KY 40506

‡. Department of Pharmacy Practice and Science, College of Pharmacy, University of Kentucky, Lexington, Kentucky, 40508

¶. Department of Cell and Molecular Physiology, Loyola University Chicago, Chicago, IL, United States

Gene Symbol	LogFC	Gene Symbol	LogFC
TNF	2.05	CD36	-5.22
IL6	4.77	ADORA3	-5.51
CXCL1 (KC/GRO)	6.14	TGFBR2	-3.77
IL1B	6	PRKCA	-3.76
IFNG	4.25	CSF1R	-3.04
CCL5	5.85	CD9	-2.9
CD54 (ICAM1)	4.95	TLR5	-4.86
TLR2	2.41	IL1R1	-3.91
TNFSF13B	2.04	CD206 (MRC1)	-4.06
CD63 (LAMP3)	6.41	GPR34	-5.73
CD40	1.67	FFAR4	-2.19
CD95 (FAS)	4.76	GNAQ	-2.55
CXCL9	7.63	CD180	-5.15
CCL1	3.52	ALOX15	-4.19
CXCL2 (MIP-2)	3.23	CCL26	-5.21

SI Table 1. Differentially expressed genes in M1 macrophage. The logFC was calculated by log(M1)-log(M2). In this case, positive logFC indicate upregulation of genes such as TNF, IL6 and IL1B in M1 and downregulation of genes CD36, ADORA3 and TGFBR2 in M1

Receptor	Score	Signaling pathway	Ligand
TLR2	5.75	’TLR2’, ’RAC1’, ’PI3K’, ’PDPK1’, ’AKT’, ’NFKB1’, ’IL1B’, ’M1 polarization’	PAMPs
CCR3	6	’CCR3’, ’GNAI1’, ’PI3K’, ’PDPK1’, ’AKT’, ’NFKB1’, ’IL1B’, ’M1 polarization’	CCL5
TLR4	6	’TLR4’, ’TICAM2’, ’TICAM1’, ’TBK1’, ’IKBKE’, ’IRF3’, ’M1 polarization’	PAMPs
CCR1	6	’CCR1’, ’GNAI1’, ’PI3K’, ’PDPK1’, ’AKT’, ’NFKB1’, ’IL1B’, ’M1 polarization’	CCL3, CCL5
CCR5	6	’CCR5’, ’GNAI1’, ’PI3K’, ’PDPK1’, ’AKT’, ’NFKB1’, ’IL1B’, ’M1 polarization’	CCL3, CCL4, CCL5
TLR1	6	’TLR1’, ’RAC1’, ’PI3K’, ’PDPK1’, ’AKT’, ’NFKB1’, ’IL1B’, ’M1 polarization’	PAMPs
TNFRSF1B	7	’TNFRSF1B’, ’TRAF2’, ’RIPK1’, ’MAP3K1’, ’MAP2K7’, ’MAPK8’, ’IRF3’, ’M1 polarization’	TNF-α
ITGAL	7	'ITGAL', 'PTK2B', 'RAC1', 'PI3K', 'PDPK1', 'AKT', 'NFKB1', 'IL1B', 'M1 polarization'	ICAM1
ITGB2	7	'ITGB2', 'PTK2B', 'RAC1', 'PI3K', 'PDPK1', 'AKT', 'NFKB1', 'IL1B', 'M1 polarization'	ICAM1
TNFRSF1A	7.5	’TNFRSF1A’, ’TRADD’, ’FADD’, ’RIPK1’, ’MAP3K1’, ’MAP2K7’, ’MAPK8’, ’IRF3’, ’M1 polarization’	TNF-α

SI Table 2. M1 polarization pathways and their associated membrane receptors identified by the approach outlined in figure S8. Pathways are shown in figure 8 for clarity.

Serial No.	Macrophage-Engineered Vesicles	Zeta Potential (mV)
1	MEVs	-5.2
2	P(TNF)-MEVs	-9.2
3	P(CD54)-MEVs	-5.5
4	pam-MEVs	-16.8
5	cpg-MEVs	-9.3

SI Table 3. Zeta potential values measured for various programmed vesicles. Negative zeta potential values of these programmed vesicles indicate improved stability in aqueous solution.

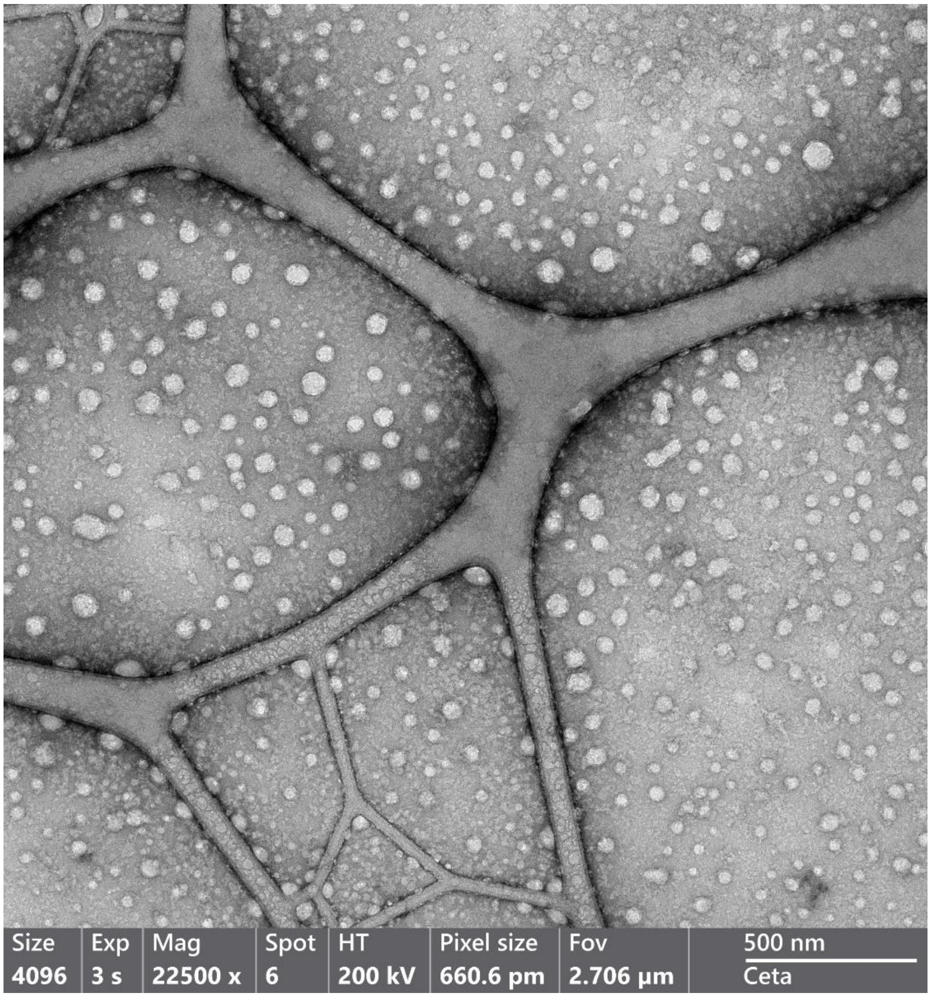

Figure S1. Transmission electron microscopy micrograph of negatively stained Raw 264.7-derived vesicles. Vesicles were measured to have a diameter ranging from 25-150 nm, with an average diameter of 70 ± 20 nm. Vesicle were observed to primarily possess a relatively spherical or oblong morphology. Deformation of the vesicles may be the result of sample preparation for TEM.

a

Cytokines	[mev] pg/mL	[mev-FR] pg/mL
IFN-γ	3±0.5	4±0.2
IL-10	16±1	17±4
IL-12p70	12±1	11±3
IL-1β	4±1	4±1
IL-6	28±6	23±6
KC/GRO	3±0.2	3±0.2
TNF-α	123±16	103±29

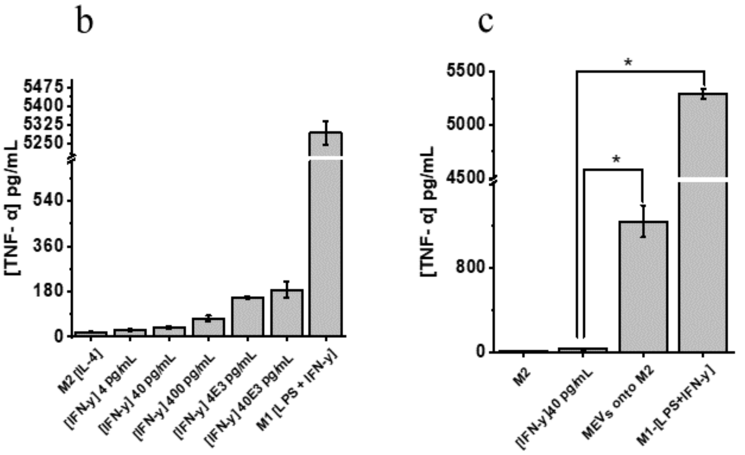

Figure S2. Cytokine content of MEVs (a) Quantification of pro-inflammatory cytokine present on MEVs. MEV were freeze-ruptured (MEV-FR) in liquid nitrogen to assess cytokines present inside of the MEVs. (b)A dose response study showing the pro-inflammatory cytokine-TNF-α released by M2 macrophages after incubation with different concentration of IFN-γ for 24 hours. (b) Quantification of pro-inflammatory cytokine-TNF-α released by M2 macrophages after incubation with 40pg/mL IFN-γ which is representative of the cytokine present on $1x10^{11}$ MEVs , $1x10^{11}$ MEVs and M1 polarizing stimulants (LPS (20 ng/mL) + IFN-γ (20 ng/mL)) for 24 hours. Supernatants were assayed in triplicate using a mouse TNF-α V-PLEX cytokine assay kit from Meso Scale Discovery.

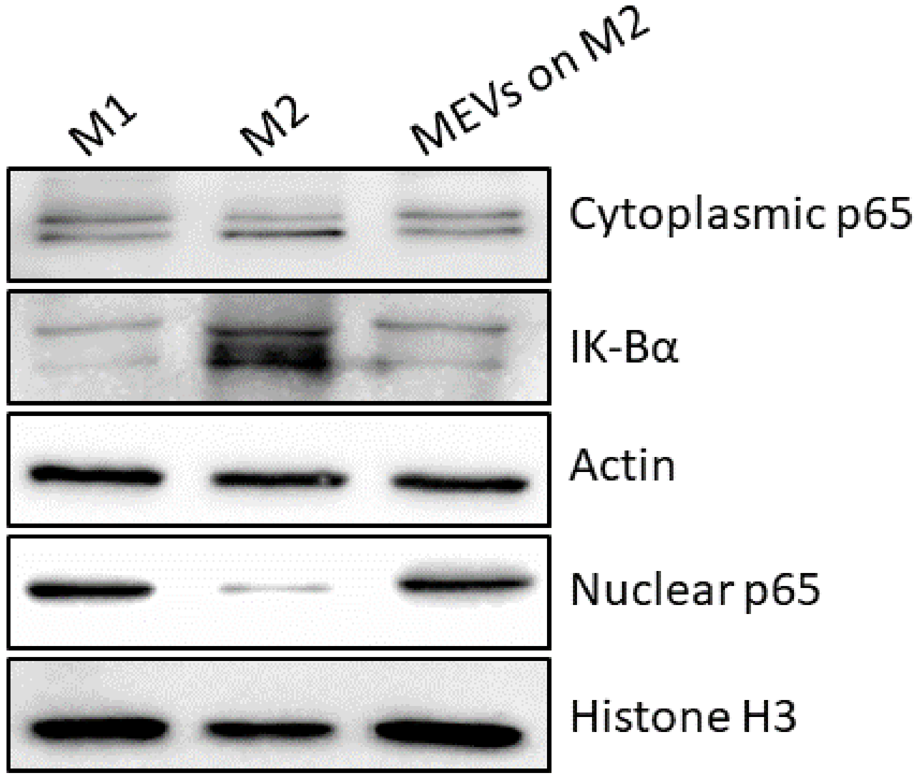

Figure S3. Western blot analysis of M1and M2 macrophages compared to MEV treated M2 macrophages for p65 translocation.

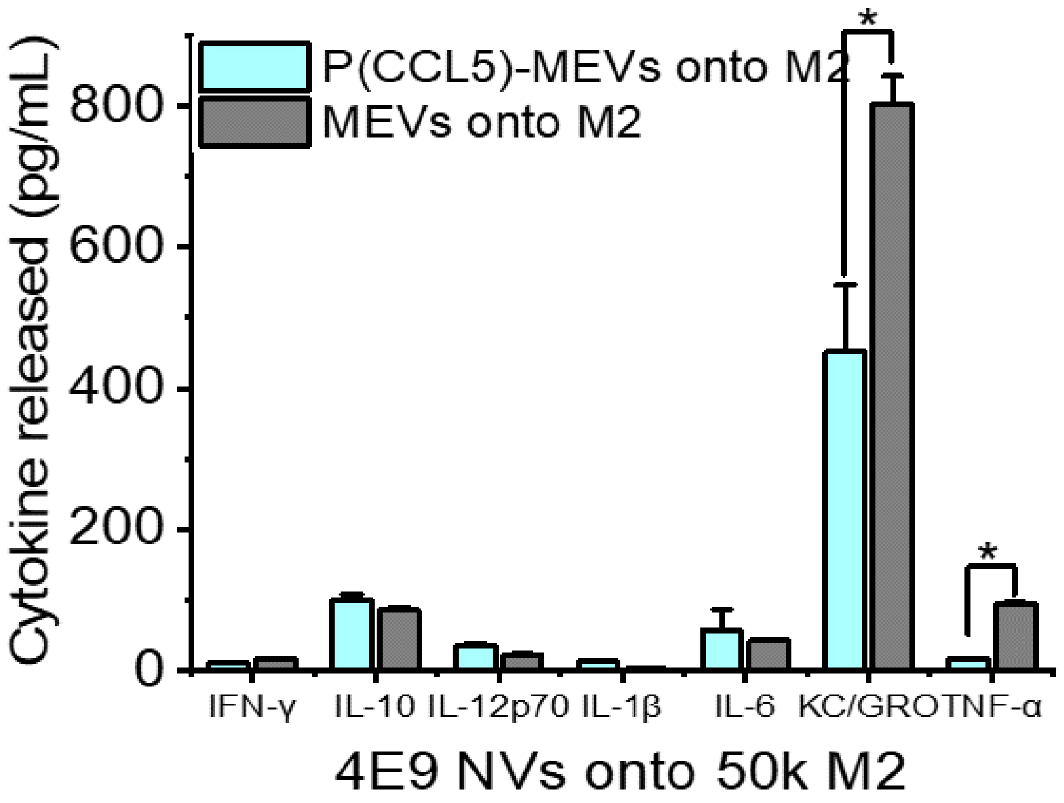

Figure S4. CCL5-programmed MEVs treated M2 BMDMs produce higher TNF-α compared M2 BMDMs incubated with regular MEVs.

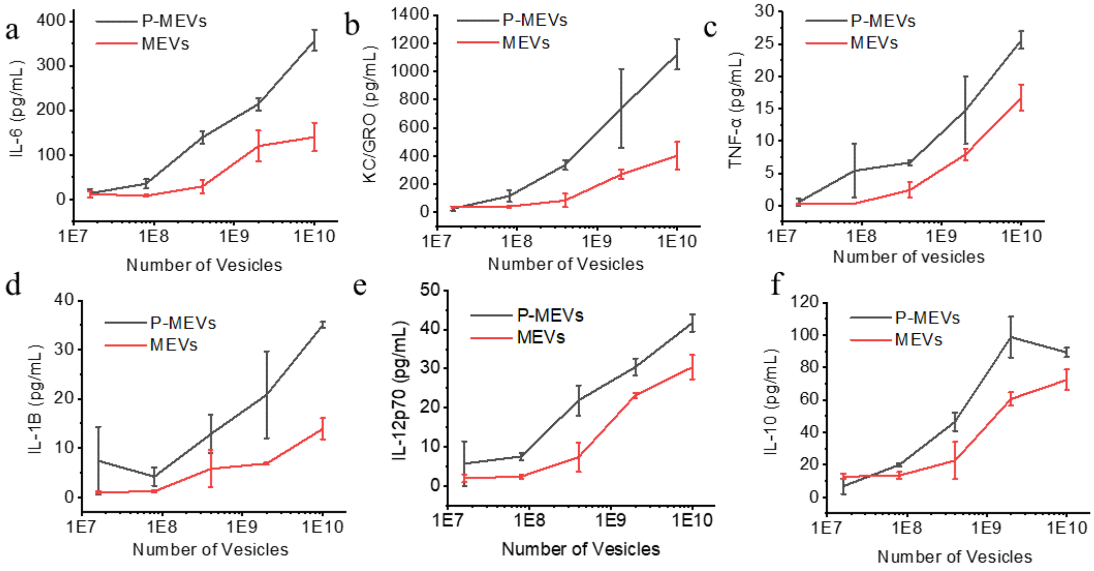

Figure S5. Dose response studies for the cytokine released by M2 macrophages that had been incubated with an increasing concentration of MEVs or CD54-programmed MEVs.

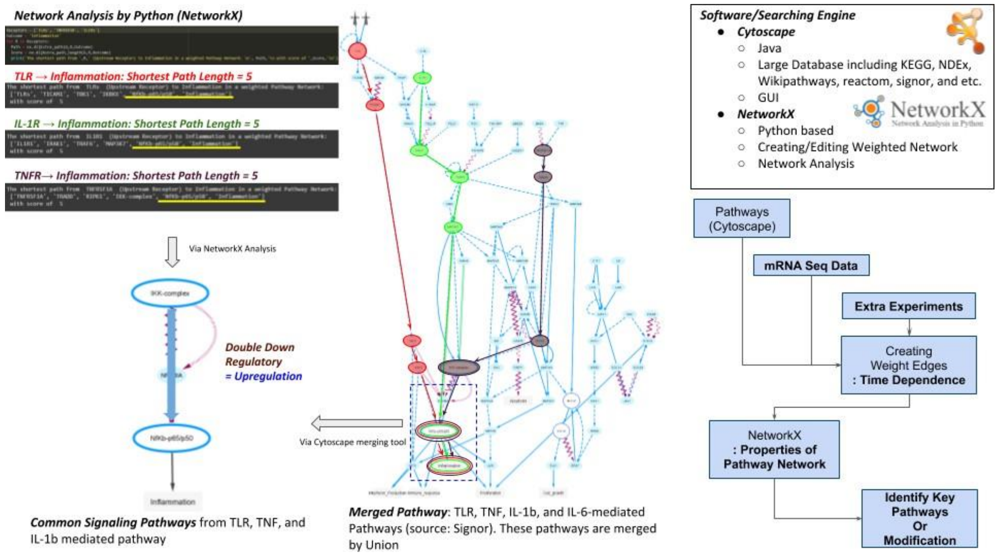

Figure S6. Summary of Cytoscape/NetworkX based network analysis.

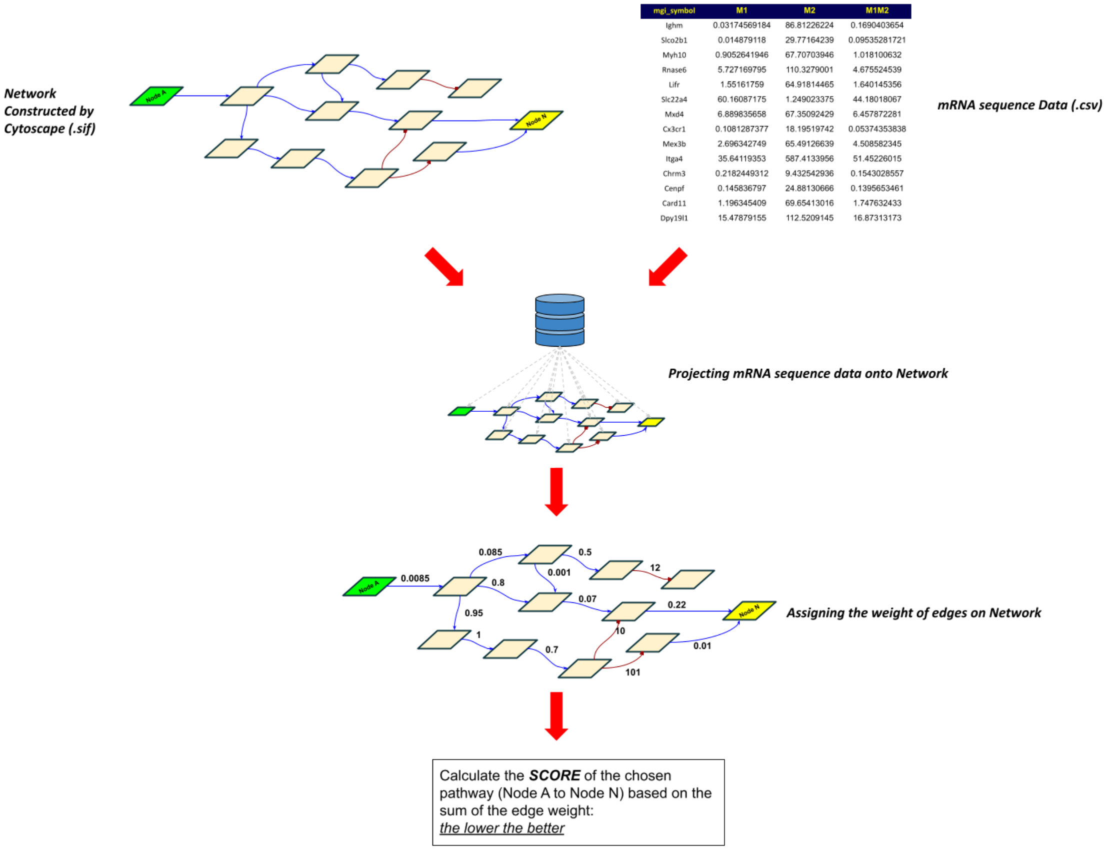

mgi_symbol	M1	M2	M1M2
Ighm	0.03174569184	86.81226224	0.1690403654
Slco2b1	0.014879118	29.77164239	0.09535281721
Myh10	0.9052641946	67.70703946	1.018100632
Rnase6	5.727169795	110.3279001	4.675524539
Lifr	1.55161759	64.91814465	1.640145356
Slc22a4	60.16087175	1.249023375	44.18018067
Mxd4	6.889835658	67.35092429	6.457872281
Cx3cr1	0.1081287377	18.19519742	0.05374353838
Mex3b	2.696342749	65.49126639	4.508582345
Itga4	35.64119353	587.4133956	51.45226015
Chrm3	0.2182449312	9.432542936	0.1543028557
Cenpf	0.145836797	24.88130666	0.1395653461
Card11	1.196345409	69.65413016	1.747632433
Dpy19l1	15.47879155	112.5209145	16.87313173

Figure S7. Demonstrating how mRNA sequence data is incorporated with the network constructed via Cytoscape.
